# Respiratory influence on cerebral blood flow and blood volume – A 4D flow MRI study

**DOI:** 10.1177/0271678X251316395

**Published:** 2025-01-30

**Authors:** Pontus Söderström, Anders Eklund, Nina Karalija, Britt M Andersson, Katrine Riklund, Lars Bäckman, Jan Malm, Anders Wåhlin

**Affiliations:** 1 Department of Applied Physics and Electronics, Umeå University, Umeå, Sweden; 2 Department of Diagnostics and Intervention, Biomedical Engineering and Radiation Physics, Umeå University, Umeå, Sweden; 3 Umeå Center for Functional Brain Imaging (UFBI), Umeå University, Umeå, Sweden; 4 Department of Medical and Translational Biology, Umeå University, Umeå, Sweden; 5 Department of Diagnostics and Intervention, Diagnostic Radiology, Umeå University, Umeå, Sweden; 6 Aging Research Center, Karolinska Institutet & Stockholm University, Stockholm, Sweden; 7 Department of Clinical Science, Neurosciences, Umeå University, Umeå, Sweden

**Keywords:** Cerebral blood flow, gating, glymphatic system, respiration, 4D flow MRI

## Abstract

Variations in cerebral blood flow and blood volume interact with intracranial pressure and cerebrospinal fluid dynamics, all of which play a crucial role in brain homeostasis. A key physiological modulator is respiration, but its impact on cerebral blood flow and volume has not been thoroughly investigated. Here we used 4D flow MRI in a population-based sample of 65 participants (mean age = 75 ± 1) to quantify these effects. Two gating approaches were considered, one using respiratory-phase and the other using respiratory-time (i.e. raw time in the cycle). For both gating methods, the arterial inflow was significantly larger during exhalation compared to inhalation, whereas the venous outflow was significantly larger during inhalation compared to exhalation. The cerebral blood volume variation per respiratory cycle was 0.83 [0.62, 1.13] ml for respiratory-phase gating and 0.78 [0.59, 1.02] ml for respiratory-time gating. For comparison, the volume variation of the cardiac cycle was 1.01 [0.80, 1.30] ml. Taken together, our results clearly demonstrate respiratory influences on cerebral blood flow. The corresponding vascular volume variations appear to be of the same order of magnitude as those of the cardiac cycle, highlighting respiration as an important modulator of cerebral blood flow and blood volume.

## Introduction

Understanding respiratory influences on cerebral blood flow and cerebral blood volume is crucial, as these variations are believed to play a vital role in maintaining brain homeostasis.^1^ Disruptions in this coupling could serve as markers or even contributors to neurodegenerative diseases.^2,3^ The Monro-Kellie doctrine highlights how changes in vascular volume interact with intracranial pressure and cerebrospinal fluid (CSF) flow,^4^ both of which are fundamental to brain homeostasis.^5^ Additionally, respiratory-induced pulsations have been suggested as potential drivers of interstitial fluid perfusion,^6–8^ particularly through venous pulsations and cyclic variations in venous blood volume, which are likely critical for perivenous outflow.^9,10^ Together, these mechanisms emphasize the significance of respiratory dynamics in cerebrovascular regulation and neurological health. Despite this hypothesized central role of the respiratory cycle, its impact on variations in total cerebral blood flow and volume have not been well studied. Importantly, the pioneering studies investigating respiratory effects on cerebral blood flow and CSF dynamics typically utilize paced breathing,^6,11,12^ which has been shown to change the power spectrum in CSF and blood flow compared to free breathing.^12^ Another pioneering approach has demonstrated differences in *cardiac-resolved* flow profiles between different respiratory phases (e.g. inhale vs exhale).^13,14^ Thus, although there are evidence supporting an important role of the respiratory cycle, the magnitude of such effects during free breathing remains largely unknown, especially regarding cerebral blood volume variations.

4D flow MRI is a non-invasive technique used to quantify flow rates in arteries and veins.^15^ The technique provides time-resolved velocities in any spatial direction through retrospective gating, which resolves the data into multiple temporal frames, usually cardiac-gated frames.^16^ The flexibility of 4D flow MRI has made it possible to extract several hemodynamic markers from cardiac-gated data, such as pulse wave velocity,^17^ arterial and venous pulsatility,^18^ and wall shear stress.^19^ The technique is not restricted to a specific gating type, and respiratory based gatings for 4D flow MRI have been evaluated in small samples by binning the data into inhale and exhale phases.^20^ However, challenges with respiratory gating when using traditional gating approaches based on time are the asymmetrical shape of the respiration cycle, (e.g. inhalation duration surpasses that of expiration) and relatively flat peaks, which can lead to ambiguities regarding what breathing phase a reconstructed respiratory time frame represents. A more robust alternative may be to transform the respiratory signal into its corresponding phase in the respiratory cycle based on the shape of the signal rather than raw time in the respiratory cycle.

The aim of this study was to investigate respiratory effects on arterial and venous cerebral blood flow and determine the corresponding variation in total intracranial vascular volume. We also compared respiration-induced variations to cardiac-induced pulsatility. To accomplish these objectives, we employed two 4D flow MRI gating approaches that bin acquired raw data according to phase of the respiratory cycle or using time since last inhalation peak.

## Materials and methods

### Participants

The participants was a subgroup from the Cognition, Brain, and Aging (COBRA) study.^21^ During the years 2022–2023, 90 individuals (34 women, 75 ± 1 years, range 73–77 years) who were originally selected randomly from the population registry in Umeå, Sweden, successfully completed the 4D flow MRI examination (second follow-up of the COBRA sample that was initiated approximately a decade earlier). We excluded data from 25 individuals due to questionable quality of the respiratory bellows signal. The final study sample thus consisted of 65 individuals. One reason for the high exclusion rate was likely that respiratory bellows were used for monitoring purposes and scans were not reperformed in episodes of poor sensor data.

The study was conducted in accordance with the Declaration of Helsinki and was approved by the ethical review board of Umeå University (approval: 2012-57-31 M). Oral and written informed consent was provided from all participants prior to any testing.

### MRI protocol

MRI acquisitions, including 4D flow MRI scans were performed on all participants using a 3T scanner (SIGNA Premier; GE Healthcare, Milwaukee, Wisconsin), with a 48-channel head coil. No contrast agents were used for the MRI acquisition, and respiratory bellows were used to track the participants’ respiration during the whole scanning period. Full-brain covering 4D flow MRI data were collected using phase contrast vastly under sampled isotropic projection reconstruction (PC-VIPR)^22^ with 5-point velocity encoding^23^ in approximately 10 minutes. The 4D flow MRI acquisition used the following imaging parameters: repetition time/echo time (TR/TE) 7.6/2.5 ms, flip angle 8°, velocity encoding (*v_enc_*) 110 cm/s, radial projections 16000, acquisition matrix 320 × 320 × 320, and imaging volume 22 × 22 × 22 cm^3^.

### 4D Flow MRI processing

The 4D flow MRI data were reconstructed into 0.69 mm isotropic voxel size. Moreover, the data were reconstructed into 20 time frames over the respiratory cycle and cardiac cycle separately, with view sharing via tornado filtering.^24^ The reconstructed velocities were corrected for Maxwell term phase offset^25^ and background phase using a polynomial of degree 3. To highlight vascular structure, an angiogram was calculated using the piecewise complex difference defined as:

(1) CDp = M sin⁡π vvenc for v <12vencM      otherwise

where *M* is the magnitude image, and *v* is the absolute velocity. Flow rates inside the vascular tree were quantified with a semi-automatic centerline-based algorithm for 4D flow MRI data.^26^ Here, the algorithm extracts centerlines from the vascular tree and determines the direction of each centerline using the third consecutive centerline-point on each side. Cut planes perpendicular to the vessels were automatically positioned along the centerlines, and linear interpolation was used to increase the resolution in the cut planes with a factor 2. For each cut plane, vessel segments were constructed using *CD_p_*, with a local intensity threshold (20% of the peak value within the cut plane). The flow rates in each vessel fragment were calculated by computing a scalar product between the direction of the centerline and the velocity vector, multiplied by the size of one voxel, summed over all voxels within the segmented vessel fragment. Then, the average flow in 15 consecutive vessel segments were used to calculate the flow rate in each vessel.

### Estimation of cerebral blood volume variations

**Figure 1.**
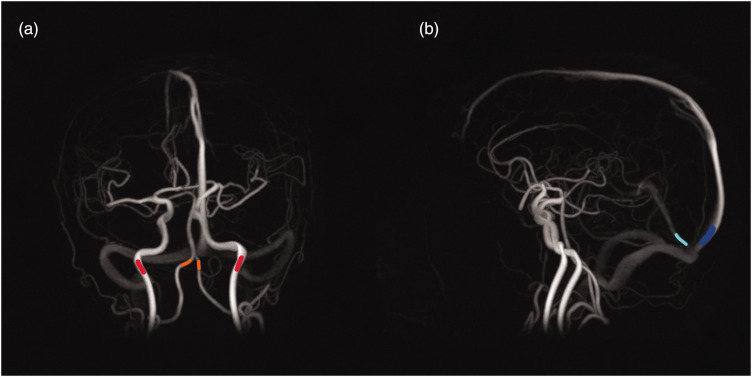
Location of flow measurements marked in angiograms from 4D flow MRI data. (a) Example of a location where arterial flow is measured. Internal carotid arteries are marked in red and vertebral arteries are marked in orange and (b) Example of a location where venous flow is measured. Superior sagittal sinus is marked in blue and straight sinus is marked in cyan.

Flow rates were extracted in the internal carotid arteries (ICAs) and vertebral arteries (VAs) on the arterial side, representing the total cerebral blood flow (tCBF). On the venous side, flow rates were extracted in the superior sagittal sinus (SSS) and straight sinus (STR), see Figure 1 for an example. The vascular volume variations related to the respiratory and cardiac cycle were estimated using the cumulative integral of the flow differences between arteries and veins normalized to a common scale:

(2) $$V_{i}\;=\;\int_{0}^{t_{i}}\left(Q_{A}\left(\tau \right)-\;Q_{V}\left(\tau \right)\frac{\;\bar{Q_{A}}\;}{\;\bar{Q_{V}}\;}\right)\text{d}\tau$$

where $Q_{A}(t)$ and $Q_{V}(t)$ are the flow profiles in the arteries (ICA + VA) and the veins (SSS + STR) respectively, $t_{i}$ is the mean time at the i^th^ respiratory or cardiac time frame, and a bar above any variable indicates means over all temporal frames. The measured venous outflow is lower than tCBF, indicating that SSS and STR only capture a portion of the total venous outflow. However, in this study we assumed that the variations in total cerebral outflow are proportional to the blood flow variations in SSS and STR. Thus, we scaled the measured venous flow with a constant factor, such that the total venous outflow matches tCBF, similar to what has been done in previous Phase Contrast (PC) MRI studies.^27,28^ On average, the ratio of tCBF to measured venous outflow was 1.86 in the current sample. $V_{i}\;$ was calculated by using the midpoint method. The time at each frame was estimated by counting the number of k-space spokes within the frame, multiplied with the total scanning time divided by the total number of spokes, and then divided by the total number of respiratory/cardiac cycles. From this, the cerebral blood volume variations were calculated as

**Figure 2.**
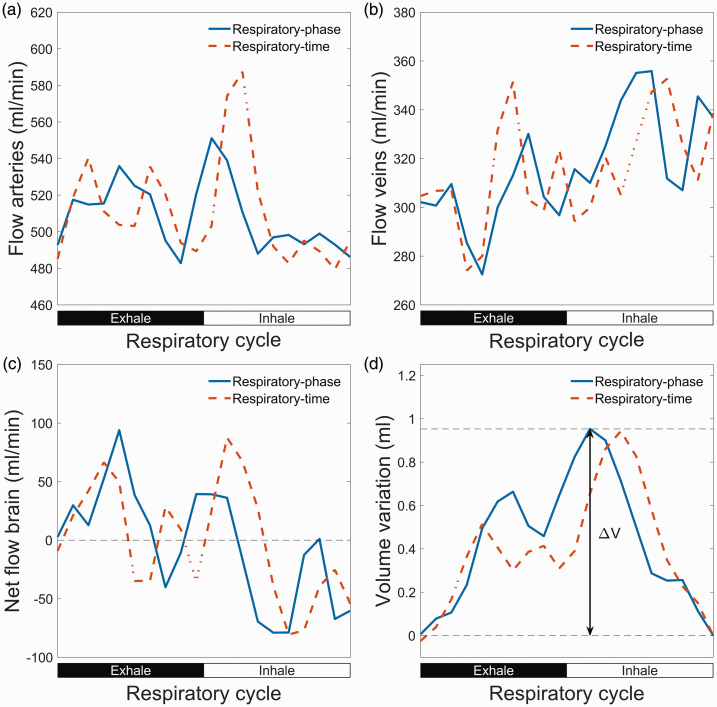
Illustration of cerebral blood volume variations over the respiratory cycle. (a) Flow rate in arteries (internal carotid arteries and vertebral arteries) over the respiratory cycle. (b) Flow rate in veins (superior sagittal sinus and straight sinus). (c) Net flow to the brain calculated as flow in arteries minus flow in veins. Here the measured venous outflow is multiplied with a constant factor so that the average venous outflow equals the average arterial inflow and (d) blood volume build-up in brain calculated through a cumulative integral of the net flow. The volume variation is the difference between maximum and the minimum of this curve. Here the volume variation is illustrated for respiratory-phase gating.

$\Delta V=\max\left(V\right)-\min\left(V\right)$ (3)

where V is the vector containing the $V_{i}$ for each respiratory or cardiac time frame. An illustration of the calculations of cerebral blood volume variations is seen in Figure 2.

### Respiratory gatings

**Figure 3.**
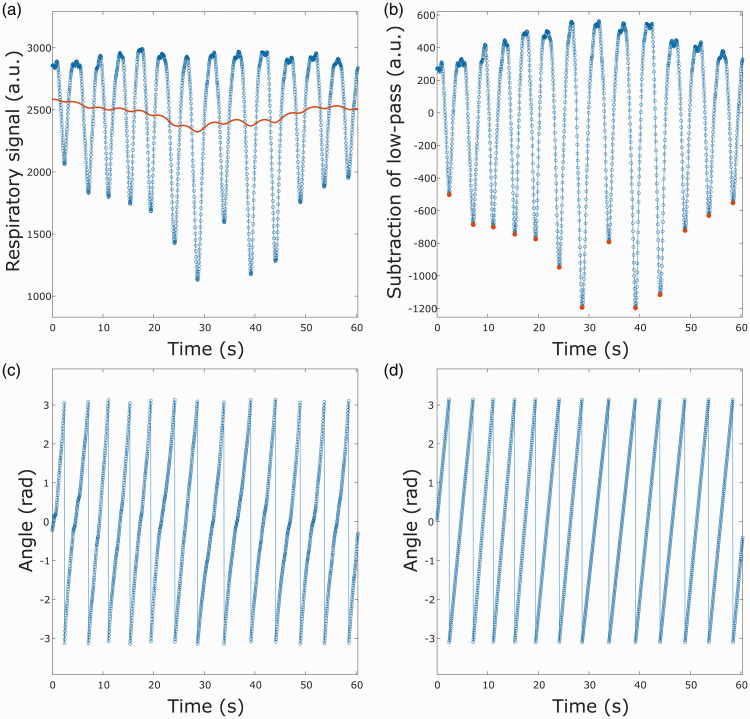
Illustration of respiratory gating steps. (a) Signal from respiratory bellows with a low-pass filter. (b) The low-pass filtered signal is subtracted from the respiratory signal and maximum inhalation peaks are marked in red. (c) Complex argument of the Hilbert transformation applied to the filtered respiratory signal and (d) respiratory gating that is linear in time from last inhalation peak.

Two respiratory-gating methods were utilized, both based on the signal from the respiratory bellows sampled during the MRI acquisition. In respiratory-phase gating, the signal was split into multiple time frames using the Hilbert-Transform (HT). The HT of a real valued signal appends an imaginary duplicate of the signal in which all frequency components are phase-shifted by ± π /2 radians. This delay between the real and imaginary part of the transformed signal allowed us to extract the corresponding phase of the raw signal using the complex argument, which has previously been used in other applications.^29,30^ In this HT-based gating method, a low-pass filtered version of the gating signal was calculated using a Butterworth filter of order 1, where the passband edge frequency and stopband edge frequency were set to 0.01 Hz and 10 Hz, respectively. The low-pass signal was then subtracted from the gating signal prior to the application of HT to center the gating signal of each respiratory cycle around zero. After that, the complex argument of the transformed signal was calculated and used as a new gating signal, with values between − π and π , where ±π indicates an inhalation peak, whereas 0 decodes to an exhalation peak. In the time-based gating method, entitled respiratory-time gating, the spokes were divided into equispaced frames based on the time since the last inhalation peak. The inhalation peaks were estimated from the HT gating signal, by finding all spokes where the phase ranges from π to − π. The peaks were further detected by searching for the minimum respiratory bellows signal in a small region around the estimated peak. The gating steps for each of the two respiratory gatings are seen in Figure 3.

**Figure 4.**
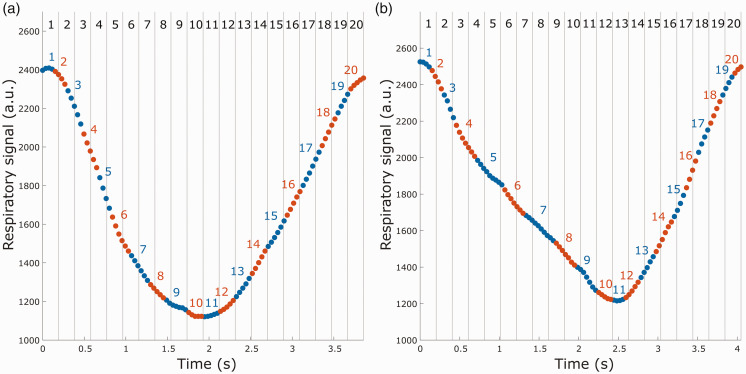
Comparison of spoke binning for the two different respiratory gatings. The dots represent spokes for one encode, and alternating colors indicate shifting frame in the respiratory-phase gating. Vertical lines represent frame division for the respiratory-time gating. (a) Frame binning for a relatively symmetrical breathing cycle and (b) frame binning for a breathing cycle with longer exhalation than inhalation.

To illustrate the differences between the two proposed respiratory-gating methods, we extracted two different breathing waveforms, as seen in Figure 4. In the first breathing cycle (Figure 4(a)), the inhale and exhale are approximately equally long, whereas in the second breathing cycle (Figure 4(b)) inhalation is shorter than exhalation. For the HT gating, the exhalation peaks occur around frame 10 and 11 in both breathing cycles. This is also the case for the respiratory-time gating for the symmetrical breathing cycle, but in the asymmetrical cycle the exhalation peak occurs around frame 12 and 13.

### Statistical analysis

All statistical analyses were performed in MATLAB (version 9.13.0. Natick, Massachusetts: The MathWorks Inc.). Reported variables were tested for normality using skewness and kurtosis, by calculating their z-score and taking z values within ±3.29 to be normal.^31^ Normal variables are reported with mean ±standard deviation, and non-normal variables are reported with median and interquartile range. All cerebral blood volume variations are presented with median and interquartile range, although the blood volume variations over the cardiac cycle was normally distributed. To compare cerebral blood volume variations between the two respiratory gatings, a paired-sample t-test was conducted. A one-sample t-test was used to evaluate the flow difference between inhalation and exhalation for both arterial and venous blood flow, whereas any difference in mean inspiratory heart rate and expiratory heart rate was tested using a paired-sample t-test. Pearson correlation coefficient was used to measure associations between two variables. Blood flow continuity within each measured vessel segment was tested through a one-way ANOVA for the respiratory-time gating, since this gating eliminates any bias from differences in k-space data per frame. Here we calculated the standard deviation of the blood flow across the 15 cut-planes used to extract the waveform for each vessel segment. Each temporal frame constitutes a group, with each subject contributing a single measurement per frame for the one-way ANOVA. P-values were considered significant at the 0.05 level when comparing gating methods. For investigations of flow rate characteristics during inhale vs exhale we made 2 tests for 2 different gatings, thus a Bonferroni adjusted significance threshold of 0.05/4 = 0.0125 was used.

## Results

### Respiratory influence on cerebral arterial and venous flow

At the individual level, the respiratory flow modulations on the arterial and venous flow differed in amplitude between the two gating methods, although the overall pattern was the same. Both gating methods showed increased arterial flow and decreased venous flow during exhalation, as well as a decreased arterial flow and increased venous flow during inhalation. For absolute values, see Table 1.

**Table 1.** Arterial (mean of internal carotid arteries and vertebral arteries) and venous (mean of superior sagittal sinus and straight sinus) flow during exhale and inhale, together with the differences in flow between inhale and exhale for the two gating methods.

	Mean arterial flow (ml/min)				Mean venous flow (ml/min)			
Gating	Inhale	Exhale	Inhale - Exhale	p-value	Inhale	Exhale	Inhale - Exhale	p-value
Phase	606 ± 93	622 ± 95	−16.4 ± 14.0	<0.001	341 ± 67	334 ± 66	7.0 ± 17.4	0.002
Time	607 ± 93	622 ± 94	−14.7 ± 14.5	<0.001	341 ± 67	333 ± 66	8.0 ± 16.4	<0.001

### Respiratory influence on cerebral vascular volume

**Figure 5.**
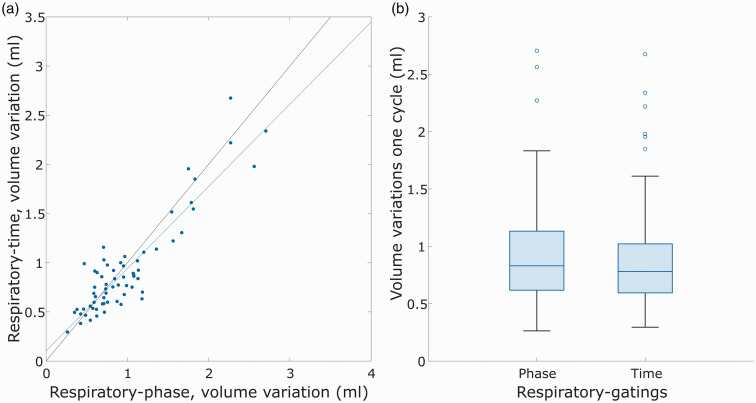
Volume variations for two different kinds of respiratory gatings. (a) Comparison of volume variations over the respiratory cycle for respiratory-phase gating vs respiratory-time gating and (b) boxplot of cerebral blood volume variations in the respiratory cycle.

Using the net difference between arterial inflow and venous outflow, we could estimate a cyclic variation in the volume of the cerebral vascular bed corresponding to 0.83 [0.62, 1.13] ml for the respiratory-phase gating and 0.78 [0.59, 1.02] ml for the respiratory-time gating. Although the derived volume variations of the two gating methods were highly related (R = 0.91, P < 0.001, Figure 5(a)), the respiratory-time gating approach systematically generated lower values (P = 0.045). A box chart of the blood volume variations for both respiratory gatings is seen in Figure 5(b). For comparison, we also calculated the cerebral blood volume variation over the cardiac cycle to 1.01 [0.80, 1.30] ml, suggesting that the volumes of respiratory and cardiac pulsations were similar in magnitude.

### Vascular volume variations on group average curves

**Figure 6.**
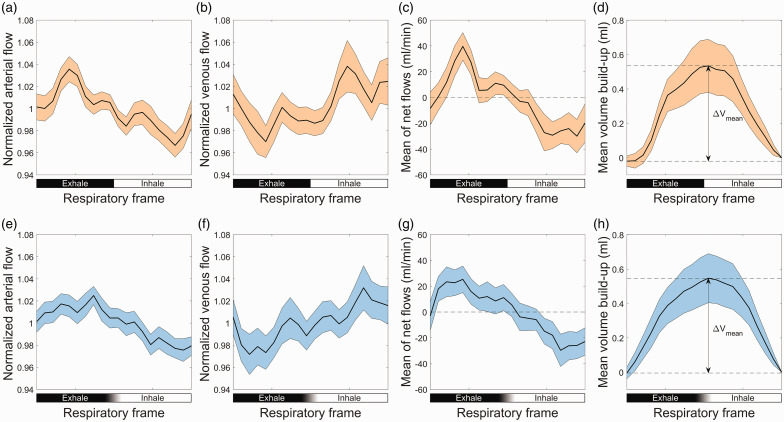
(a) Average waveforms in arteries over the respiratory cycle for respiratory-phase gating. Individual waveforms are normalized by division of its mean flow. (b) Average waveforms in veins over the respiratory cycle for respiratory-phase gating. Individual waveforms are normalized by division of its mean flow. (c) Average net flow to the brain for respiratory-phase gating. (d) Average volume build-up for respiratory-phase gating. (e) Average waveforms in arteries over the respiratory cycle for respiratory-time gating. Individual waveforms are normalized by division of its mean flow. (f) Average waveforms in veins over the respiratory cycle for respiratory-time gating. Individual waveforms are normalized by division of its mean flow. (g) Average net flow to the brain for respiratory-time gating and (h) average volume build-up for respiratory-time gating. The error-bars for all plots are given as 95% confidence interval for the mean, with ±2SD/ $\sqrt{n}$ .

Group average respiratory modulations of normalized flow curves are visualized in Figure 6. Both respiratory-phase and respiratory-time based retrospective binning of the 4D flow MRI data detected significant blood flow modulations on both the arterial inflow as well as the venous outflow, see Figure 6 subpanels (a), (b), (e) and (f). The average net flow curve revealed a dependence on the respiratory cycle, corresponding to a respiratory-dependent volume change of the cerebral vasculature of 0.55 ml for both respiratory gating approaches.

### Effect of motion

Since potential respiratory-cycle synchronized motion could create artificial modulations in the flow rate we investigated both potential heart rate differences, and respiratory-frame dependent changes in flow rate variability between consecutive cross sections. There was no significant difference between the mean inspiratory heart rate and the mean expiratory heart rate in this cohort (P = 0.18). Moreover, there was no difference between respiratory frames with regards to inter-cross-section variability along the vessel segments, neither in arteries (P = 0.77) nor veins (P = 0.81).

## Discussion

Using 4D flow MRI we described the cyclic variations in cerebral blood flow and blood volume during normal breathing in older individuals. The observed respiratory-cycle dependent volume variation in the cerebral vasculature was comparable to that of the cardiac cycle, thus establishing normal respiration as another potentially important source of CSF redistributions within the craniospinal compartment. This observation on the cerebral vasculature extends previously described redistributions of CSF that occur during paced deep breathing.^10^ Intra-cranial pressure recordings have shown that the pressure-gradient amplitude of respiratory modulations is ∼3 times lower than that of the cardiac cycle, although CSF redistributions in the cerebral aqueduct appear dominated by respiration.^32^ This highlights that, with a slower cycle, momentum is allowed to build up over a longer period of time.^32^ Similarly, measurements of CSF flow in sylvian aqueduct and foramen magnum have revealed that the respiratory component of the CSF displacement is larger than the cardiac component, although the cardiac component of the CSF velocity is larger than the respiratory component.^11^

In this study, we measured the blood volume variations per respiratory and cardiac cycle. According to the Monro-Kellie doctrine, changes in intracranial cerebral blood volume should be compensated by an equally large CSF volume.^4^ With that simple perspective applied on our results, one can imagine that respirations lead to identical CSF movements but only at a fourth of the pace to that of the cardiac cycle. However, CSF flow measurements at the cranio-cervical junction have showed that respiratory induced CSF volume displacements are larger,^33^ potentially due to the momentum differences between the cycles. Another important aspect is that the cardiac and respiratory cycles likely have different penetration depths and therefore different relative contributions in the arterial and venous vascular levels (e.g. arterial, arteriolar, capillary, venular, venous) that experience the volume change. It is therefore conceivable that cardiac and respiratory cycles have different roles in CSF redistributions and brain clearance (e.g. cardiac more important for inflow and respiration more important for outflow).

The selection to synchronize the data according to phase or time of the respiratory cycle influenced the derived flow curves. Specifically, difficulties in peak localization and within-subject variability in the shape of the respiratory cycle may have led to an underestimation of the respiratory influences on the studied parameters (average flows and volume change) for the respiratory-time method. The differences can be visually appreciated in Figure 6, as more flat appearance of the time-gated flow curves in subpanels (e)–(g) as compared to the phase-gated counterparts in subpanels (a)–(c). We believe that considering respiratory-phase as the gating variable, mitigated these issues and allowed a more representative description of the respiratory effects on cerebral blood flow and volume. Moreover, the data could have been reconstructed based on the amplitude of the respiratory bellows signal, similar to an approach that was investigated in a previous study of cardiorespiratory double-gatings.^20^ But such gating would suppress the physiological effects on cerebral blood flow since flow modulations were phase-shifted compared to amplitude (this can be inferred from Figure 6 where the two mid-amplitude flow rates – occurring approximately in the middle of the inhalation and expiration phases, are associated with opposing flow modulations). This is in line with the non-significant difference that Schrauben et al. got when comparing venous flow at inspiration plateaus with expiration plateaus.^20^

In the current study we reconstructed the data into 20 temporal frames since such amounts of data-per-frame, together with the centerline-analysis method, is validated against 2D PC MRI (for investigating cardiac cycle dynamics).^34^ Reconstructing into a lower temporal resolution was not deemed necessary since noise levels appeared low. Reconstructing into more temporal frames was not performed as high frequency modulations over the respiratory cycle were considered unlikely. An additional gating option is 5D flow MRI^35^ that separates effects from the cardiac and respiratory cycle. Therefore, 5D flow can reduce motion artifacts from both cardiac and respiratory components. However, for intracranial applications that advantage is not as striking as for cardiac applications since the respiratory and cardiac induced motions in the cerebral vasculature are in general small. Furthermore, since we studied arteries encapsulated in bone, as well as venous sinuses formed by the stiff dura, we expected cardiac-related motion contamination to be minimal, verified by sharp vasculature on complex difference reconstructions. With these considerations, we regarded it as unnecessary to split data between two cycles, and risk that too few respiratory resolved frames were available to accurately quantify cerebral blood flow and volume variations.

In a previous study using 2D PC MRI, Kollmeier et al. described smaller respiration driven dynamics during normal breathing, as compared to forced breathing,^12^ but the magnitude of these changes and their influence of cerebral blood volume were not reported. Moreover, they considered veins at cervical level that usually have higher compliance and could change more in size by variations in central venous pressure. It has been shown that SSS has about 10 times lower flow variance compared to internal jugular veins (IJV),^36^ indicating that cervical flow measurements cannot be used as a proxy to understand intracranial respiration-induced variations. Furthermore, we calculated the cerebral blood volume variation over the cardiac cycle to compare the respiratory effects to the cardiac effects. The cardiac-driven volume variations in this study were in the same order of magnitude as reported in a previous study.^37^

We introduced a novel respiratory gating method based on HT to make the gating less sensitive to asymmetrical breathing patterns. The approach of using HT to split oscillatory signals into the corresponding phase has successfully been applied to physiological signals before.^29,30^ This approach suffers the limitation that it may fail to determine an accurate phase if the signal does not have a narrow band property,^38^ meaning that the signal occupies a small range of frequencies. However, due to the relatively small variations in breathing frequencies around 0.25 Hz and that the relative change in instantaneous amplitude of the respiratory-bellows data is in general slow compared to the breathing frequency, the respiratory-phase gating is by design less affected by this limitation.

We observed that different respiratory frames had similar variability across the 15 cut-planes used to measure flow. This suggested that motion did not introduce false flow modulations between different respiratory frames, as motion has been shown to introduce higher variability of the extracted flow.^39^ This result can be understood by considering that cardiac and respiratory motion in the cerebral vessels are in general small and should therefore have a small impact on the results. Moreover, respiration may affect the main magnetic field (B_0_), which in turn can affect the flow measurements. However, respiratory effects on B_0_ at positions superior to C2-level are in general small.^40,41^

## Limitations

Limitations in this study include averaging of several respiratory cycles due to the relatively low temporal resolution for 4D flow MRI data. This could suppress the modulation of single cycles. In future studies, temporal resolution could potentially be improved using Compressed Sensing (CS) for 4D flow MRI,^42,43^ however the increased temporal resolution is typically accompanied by a reduction in spatial resolution. In addition, our measure on cerebral blood volume variation may overestimate the true physiological effect by taking the difference between the maximum and minimum of the volume build-up, that could result in an overestimation due to noise. However, the cumulative integral tends to average succeeding noise towards zero, which make the measure less sensitive to noise. An additional noise-related issue is that we here employed a velocity sensitivity setting adapted for the arterial system. However, due to the centerline-approach, in which multiple planes are averaged under the assumption of conservation of mass, the noise-levels are drastically decreased, thus compensating sub-optimal velocity-to-noise characteristics. Another limitation is that we can only measure parts of the outflowing venous blood from the brain. By normalizing the outflowing blood such that the average outflow matched the average inflow, we could still analyze differences in inflow and outflow, but the validity of this approach requires that the measured venous waveform is representative also for other outflow pathways. We measured the venous sinus blood flow upstream of the confluence of sinuses. Downstream, veins draining the brain continues to feed into the transverse sinus, as well as into the sigmoid sinus, that eventually drains into the internal jugular vein. We expect this additional flow, which was unmeasured here, to exhibit a similar waveform to the measured flow, based on observations on the widespread correlation between cerebral vein cardiac-related oscillations and corresponding oscillations in superior sagittal flow, that appears independent of which brain region that is considered for the cerebral vein measurements.^44^ The concept that measured venous flow waveforms are representative of the other pathways that are not measured is not new. It was introduced in previous examinations of cerebral volume variations during the cardiac cycle.^27,28^ Mathematical modeling together with ultrasound imaging suggest that although the blood flow response in vertebral venous plexus and intrajugular vein during the Valsalva maneuver is compensatory in upright position, the response in each route is much more similar in supine position.^45^ Therefore, although we cannot know exactly how closely the unmeasured flow follows the measured waveforms, we expect that in the supine position, the potential difference is small, and therefore the impact on our results is likely limited.

## Conclusion

In conclusion, we quantified cerebral blood flow and volume variations over the respiratory cycle using 4D flow MRI, utilizing two different types of respiratory gating methods. Our measurements showed that the cerebral blood volume variations over the respiratory cycle were of the same order of magnitude as the cerebral blood volume variations over the cardiac cycle. The cerebral blood volume variations quantified in this study can inform further analyses linking vascular pulsations to brain clearance.

## Data Availability

Data is available from the corresponding authors upon reasonable request.
